# Nuclear Factor-κB (NF-κB) Regulates the Expression of Human Testis-Enriched *Leucine-Rich Repeats and WD Repeat Domain Containing 1* (*LRWD1*) Gene

**DOI:** 10.3390/ijms14010625

**Published:** 2012-12-28

**Authors:** Yen-Ni Teng, Po-Jung Chuang, Yo-Wen Liu

**Affiliations:** 1 Department of Biological Sciences and Technology, National University of Tainan, No.33, Sec. 2, Shulin St., West Central District, Tainan City 700, Taiwan; E-Mail: amateras300@gmail.com; 2 Department of Biotechnology, Chia Nan University of Pharmacy and Science, 60 Erh-Jen Road, Sec. 1, Pao-An, Jen-Te Hsiang, Tainan 717, Taiwan; E-Mail: pojung1027@gmail.com

**Keywords:** NF-κB, LRWD1, promoter

## Abstract

The human *Leucine-rich Repeats and WD repeat Domain containing 1* (*LRWD1*) gene was originally identified by cDNA microarray as one of the genes down-regulated in the testicular tissues of patients with severe spermatogenic defects. Human LRWD1 is a testicular-enriched protein that is present predominantly in the cytoplasm of spermatocytes and spermatids and colocalizes with the centrosome at the base of sperm tail. Reporter assay, Chromatin immunoprecipitation (ChIP) analysis, and gel electrophoretic mobility shift assay (EMSA) were used to identify the core promoter region of *LRWD1*. A 198 bp segment upstream of the *LRWD1* transcription initiation site exhibited promoter activity. The *LRWD1* core promoter lacked a TATA box but contained a NF-κB binding site. Chromatin immunoprecipitation (ChIP) analysis and gel electrophoretic mobility shift assay (EMSA) showed basal binding of the NF-κB subunit to the *LRWD1* promoter. *LRWD1* promoter activity was positively regulated by NF-κB, and this regulation was dependent on the presence of the conserved κB site in the *LRWD1* promoter region. Our data suggest that NF-κB is an important regulator for the expression of *LRWD1*. This is the first study showing that the expression of the testis-enriched *LRWD1* gene is regulated by NF-κB.

## 1. Introduction

The human *Leucine-rich Repeats and WD repeat Domain containing 1* (LRWD1) gene was originally identified by cDNA microarray as one of the genes down-regulated in the testicular tissues of patients with severe spermatogenic defects [1]. It encodes a protein with a leucine-rich repeat (LRR) domain in the *N*-terminus and three tryptophan-aspartic acid (WD40) motifs in the *C*-terminus. Immunostaining of mouse testis sections detected high levels of LRWD1 in the cytoplasm of primary spermatocytes to mature spermatozoa and much weaker signals in spermatogonia [2]. On mature spermatozoa, the anti- LRWD1 antibody stained strongly the connection region between the head and the neck where the centrosome is located. In somatic cells, LRWD1 is considered as a component of the ORC and plays critical roles in the initiation of DNA replication and cell-cycle progression [3–5]. In our previous study, we detected LRWD1 in the testicular tissues of all human subjects with germ cells, but not those of SCOS patients. On human spermatozoa, LRWD1 was found to colocalize with centrin in the centrosome region. We suggest that the testis-enriched LRWD1 is an important factor in human spermiogenesis and tail formation (manuscript submitted).

Nuclear factor-κB (NF-κB) is a ubiquitous redox sensitive and DNA sequence-specific transcription factor that is assembled from two of the five known mammalian subunits (RelA/p65, RelB, c-Rel, p50, and p52) [6–8]. In most cells, NF-κB is present as a dimer of p65 and p50 in a latent/inactive form, bound to the inhibitory protein IκB in the cytoplasm. Degradation of IκB leads to the exposure of the nuclear localization signals on the p65/p50 complex, resulting in rapid translocation of NF-κB to the nucleus where it binds to specific κB recognition elements in the promoter regions of its target genes [9]. In general, NF-κB is considered a major regulator of immune and stress responses [6,10]. The NF-κB transactivating subunit RelA (p65) plays a critical role in mouse liver development because RelA −/− mice die during mid-gestation from massive hepatocyte apoptosis [11,12]. NF-κB may also regulate male germ cell apoptosis because there is growing evidence that NF-κB has a function in cell proliferation and apoptosis [10,13], and some studies suggest the involvement of NF-κB in human and other mammalian spermatogenesis [14,15]. The expression of NF-κB factors is stage specifically controlled and may play a role during the development of rat and mouse sperm cells [16,17]. In the rat testis, the NF-κB proteins are present in Sertoli cell and spermatocyte nuclei in a stage specific manner and may play a role in the regulation of stage-specific gene expression during rat spermatogenesis [17].

Pentikäinen *et al.* characterized the expression of NF-κB in human testes and its role in testicular apoptosis [15]. They first studied the constitutive expression and DNA-binding activity of the NF-κB proteins in normal adult human testes. They then explored the induction of NF-κB DNA-binding activity and nuclear translocation during human testicular apoptosis using an *in vitro* tissue culture system and evaluated the effects of NF-κB inhibition on testicular germ cell survival [15,18]. Their results indicate that during testicular stress Sertoli cell NF-κB proteins exert proapoptotic effects on germ cells, which raises the possibility that pharmacological inhibition of NF-κB could be a therapeutic strategy in transient stress situations involving excessive germ cell death. NF-κB is also sensitive to oxidants, antioxidants and conditions that affect the intracellular redox state [19]. Previous studies have shown that NF-κB plays important roles in selenium regulated spermatogenesis [20].

Few studies have focused on specific target genes regulated by NF-κB in human spermatogenesis. We have used a series of deletion constructs with a dual luciferase reporter system to identify the core promoter of LRWD1. Site-directed mutagenesis and electrophoretic mobility shift assays (EMSA) identified a NF-κB binding element within the LRWD1 core promoter. Overexpression of NF-κB in cells enhanced LRWD1 promoter activity *in vitro*. Our results suggest that NF-κB regulates the expression of LRWD1.

## 2. Results and Discussion

### 2.1. Promoter Activity in the 5′-Flanking Region of the Testis-Enriched *LRWD1* Gene

**Figure 1:**
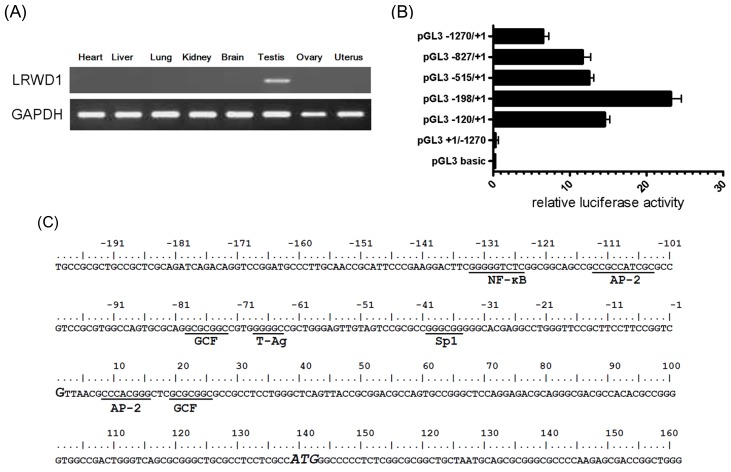

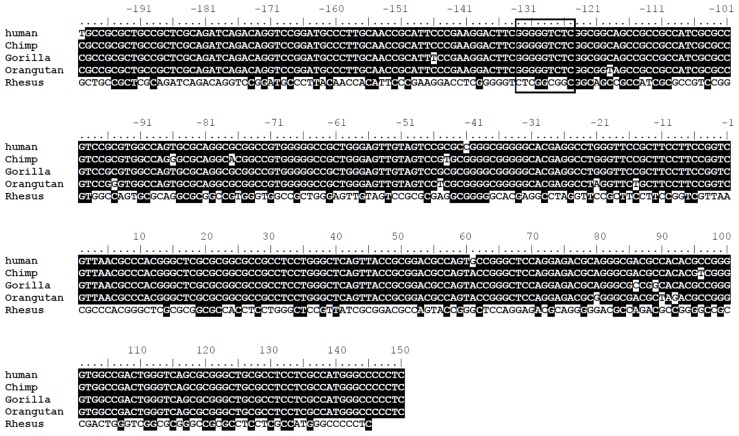
*Leucine-rich Repeats and WD repeat Domain containing 1* (*LRWD1*) expression and the *LRWD1* core promoter. (**A**) Reverse transcription- polymerase chain reaction (RT-PCR) was used to analyze the expression of *LRWD1* from Human Total RNA Panel (Clontech, Palo Alto, CA, USA). The housekeeping *Glyceraldehyde-3-phosphate dehydrogenase* (*GAPDH*) gene was used as a control; (**B**) Defining the *LRWD1* promoter. Segments spanning various length of the *LRWD1* 5′ flaking region were subcloned into the pGL3-Basic luciferase reporter vector. The transcription initiation site is assigned position +1. NT2/D1 Cells were transfected with either the empty vector or a *LRWD1* promoter reporter vector in conjunction with the control Renilla luciferase vector pRL-TK for normalization. Extracts were taken 24 h after transfection and the luciferase activity was determined. Luciferase activities were normalized to the pRL-TK reporter activities and shown as -fold induction. Means ± S.D. of triplicates from three independent experiments are shown; (**C**) Sequence of the *LRWD1* core promoter region and prospective transcriptional factor binding sites as predicted by the Promoter 2.0 Prediction Server (http://www.cbs.dtu.dk/services/Promoter/ [24]). The transcription initiation site (TSS), as determined by SMART RACE, is indicated in larger font and is assigned nucleotide +1. The binding sites of known transcription factors are underlined. The translation start codon at +139 is in larger font and italics; (**D**) Alignment of primate *LRWD1* promoter sequences. The *LRWD1* promoter regions of human (*Homo sapiens*), chimp (*Pan troglodytes*), gorilla (*Gorilla gorilla gorilla*), orangutan (*Pongo pygmaeus abelii*), and rhesus (*Macaca mulatta*) are aligned and identical nucleotides are *shaded*. As expected, human, chimp, gorilla, and orangutan *LRWD1* genes are most similar and share highest identity. The identities between human and chimp, gorilla, orangutan and rhesus are 98.0%, 98.2%, 96.8%, and 31.1%, respectively. The alignment was performed using the Clustal W Multiple alignment. The κB site (NF-κB binding site) at (−133)~(−125) is boxed.

The expression of *LRWD1* in several human tissues was examined by RT-PCR analyses on the Clontech Human Total RNA Panel. The results showed that *LRWD1* was highly and specifically expressed in the testis (Figure 1A). Our previous 5′RACE (rapid amplification of cDNA 5′ ends) mapped the transcription initiation site of *LRWD1* to nucleotide position139 upstream of the ATG start codon (manuscript submitted). In this study, we defined the *LRWD1* core promoter region by transfecting a series of deletion constructs with a dual luciferase reporter system into human testicular embryonal carcinoma NT2/D1 cells. We subcloned a fragment containing the sequence from positions −1270 to +1 (−1270/+1) and a series of 5′ deletions upstream of the firefly luciferase reporter in pGL3-Basic vector and measured luciferase activities in NT2/D1 cells transfected with the vectors. The pRL-TK vector, which contains the *Renilla* luciferase gene driven by the thymidine kinase promoter, was used as a control to normalize the transfection efficiency. Interestingly, truncation of the promoter up to −198 resulted in a substantial increase in the promoter activity (Figure 1B). Further deletion up to −120 resulted in a considerable decrease in luciferase activity, suggesting the presence of positive regulatory segments between −198 and −120 in the promoter region. Together, these findings indicate that the −198/+1upstream region of the *LRWD1* gene is important for *LRWD1* transcription activity.

### 2.2. The *LRWD1* Promoter Region Includes an Evolutionarily Conserved κB Site

Computer analysis using the PROMOTER SCAN software (http://www-bimas.cit.nih.gov/molbio/proscan/) [21] revealed several consensus binding sites for transcription factors in the LRWD1 5′-flanking sequence. There are no TATA boxes upstream of the transcription start site (TSS, +1) (Figure 1C). There are putative binding sites for Ap-2 ((−113)~(−104)), GCF ((−79)~(−73)), T-Ag ((−69)~(−64)), and Sp-1 ((−40)~(−35)) [22] within the 198 bp promoter region. Sp 1 has been shown to play a role in activating TATA-less promoters [23]. Analysis of the 1kb segment immediately upstream of the LRWD1 translational start site also revealed a κB site (GGGGGTCTC) at −133 to −125, which is a binding sequence for the NF-κB transcription factor. The sequence and location of this κB site within the promoter are highly conserved in human, chimp, gorilla, and orangutan (Figure 1D), but not in other mammals or vertebrates such as mouse, rat, or zebrafish (data not shown). Thus the κB site within the LRWD1 promoters of primates most likely plays an important role in transcriptional regulation of the gene.

### 2.3. The κB Site within the LRWD1 Promoter Binds NF-κB in Testicular Germ Cell Nuclei

**Figure 2:**
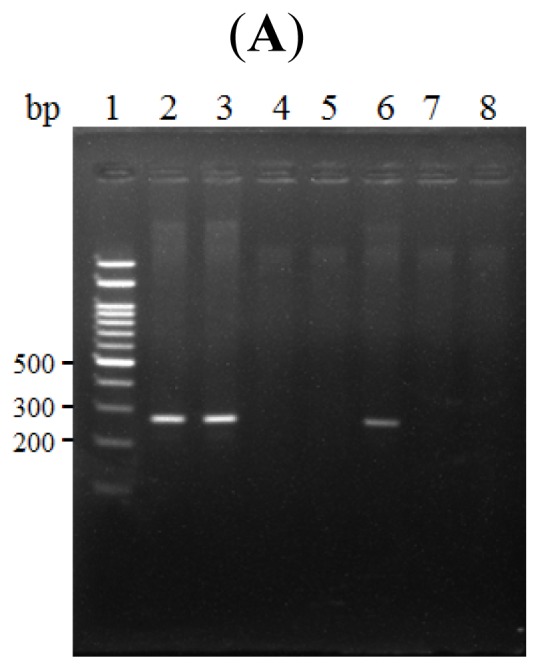

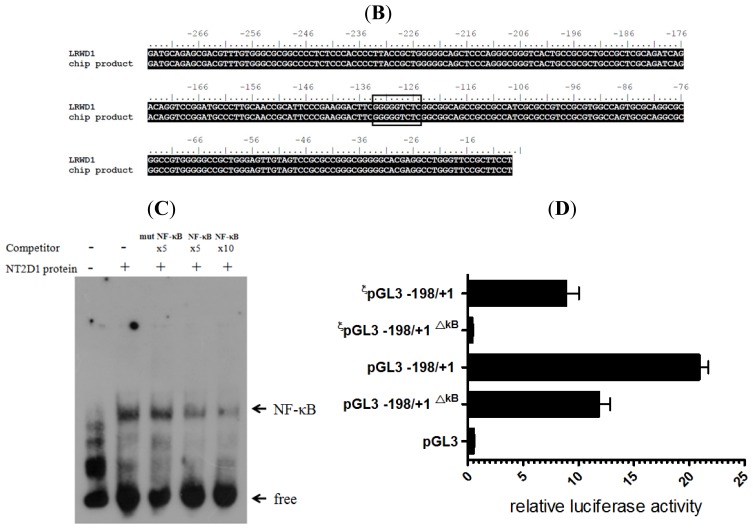
Nuclear factor-κB (NF-κB) binds to the *LRWD1* promoter. (**A**) Formaldehydecrosslinked chromatin prepared from NT2/D1 cells was immunoprecipitated (IP) with an anti-NF-κB antibody (lanes 6–8) or the control IgG (lanes 4, 5) and PCR amplified with primers specific for the *LRWD1* promoter. An aliquot of the total input DNA before immunoprecipitation served as the positive control (lanes 2, 3). As a negative control, a fragment of comparable size (up 6000) was amplified from a site about 6 kb upstream of *LRWD1* transcription start site (lanes 7, 8). Lane 1: DNA marker; Lane 2: Input DNA (before IP)-CHIP-LRWD1; Lane 3: Input DNA(before IP)-up 6000; Lane 4: IP/IgG-ChIP-LRWD1; Lane 5: IP/IgG-up6000; Lane 6: IP/p65-ChIP-LRWD1; Lanes 7, 8: IP/p65-ChIP-up6000; (**B**) Sequence alignment of the *LRWD1* promoter and the ChIP PCR product. The κB site at (−133)~(−125) is boxed; (**C**) Formation of specific DNA-protein complexes on the NF-κB sequence as detected by band-shift assays. Nuclear extracts prepared from NT2/D1 cells were subjected to band-shift assays using a labeled NF-κB oligonucleotide as a probe in the presence or absence of increasing amount of non-labeled mutated NF-κB oligonucleotides (Mut NF-κB) and non-labeled NF-κB oligonucleotides (×5, ×10) as competitors at the indicated molar ratios. Specific DNA-protein complexes are designated as NF-κB. “free” indicates the position of the free probe; (**D**) The KB site is important for *LRWD1* promoter activity. NT2/D1 cells were transiently transfected with luciferase reporter plasmids driven by either no promoter (pGL3-Basic), a 298 bp human *LRWD1* promoter fragment including (pGL3 −198/+1) or lacking (pGL3 −198/+1^ΔκB^) the κB site. The cells were also cotransfected with the pRL-TK reporter plasmids. Luciferase activities were normalized to pRL-TK reporter activities and shown as fold induction compared with the empty vector control. ξ, cells were treated with 50 μM Bay 11–7082 for 1 h after lipofection. Means ± SD of triplicates from three independent experiments were shown. ** *p* < 0.01.

We next performed chromatin immunoprecipitation (ChIP) assays to test whether NF-κB directly binds to the κB site within the LRWD1 core promoter in testicular NT2/D1 cells. NT2/D1 cell nuclei were cross-linked with formaldehyde and the lysates were immunoprecipitated with an antibody against the p65 subunit of NF-κB. The presence of the LRWD1 promoter in the DNA fraction precipitated by the antibody was PCR amplified using primers specific for the LRWD1 promoter. As shown in Figure 2A, the predicted 268 bp PCR product was amplified from samples derived from NF-κB immunoprecipitates, but not from samples immunoprecipitated with a control IgG. The sequence of the ChIP -PCR product was confirmed to be identical to the LRWD1 promoter region (Figure 2B). These data demonstrate that NF-κB interacts with the LRWD1 promoter within the NT2/D1 cell nuclei. We further tested whether transcription factor(s) in the NT2/D1 nuclear extract could bind the LRWD1 promoter by electrophoretic mobility shift assay (EMSA) using an oligonucleotide containing the −143/−115 region as the probe (Figure 2C). Multiple DNA–protein complex bands were observed using NT2/D1 nuclear extracts. The most prominent gel-shift band was eliminated when non-labeled −143/−115 oligonucleotide was used as a competitor. In contrast, a mutant competitor, in which the GGGGGTCTC sequence of the κB site was changed to GaaaagagC, could not compete for the complex formation. These results indicate that the testicular nuclear protein binds directly to the κB site which is a well-established binding site for NF-κB.

### 2.4. NF-κB Binds to the LRWD1 Promoter and Regulates Its Activity

Thus far, we have shown that NF-κB binds the *LRWD1* promoter. Because the mechanism of action of the NF-κB family members is through transcriptional modulation of target genes, we further tested whether NF-κB directly regulates *LRWD1* transcription. We generated two luciferase reporter constructs in pGL3, one contained the −198/+1 segment with the wild-type sequence (pGL3 −198/+1), and the other had deletion of the κB site (pGL3 −198/+1^ΔkB^). As shown in Figure 2D, NT2/D1 cells transfected with pGL3 −198/+1^ΔkB^ had more than 40% reduction in luciferase activities compared to cells transfected with pGL3 −198/+1. These data indicate that full basal *LRWD1* promoter activity in NT2/D1 cells requires the conserved κB site which is essential for the binding of the NF-κB transcription factor.

### 2.5. Activation of NF-κB with LPS Increases LRWD1 Expression

**Figure 3:**
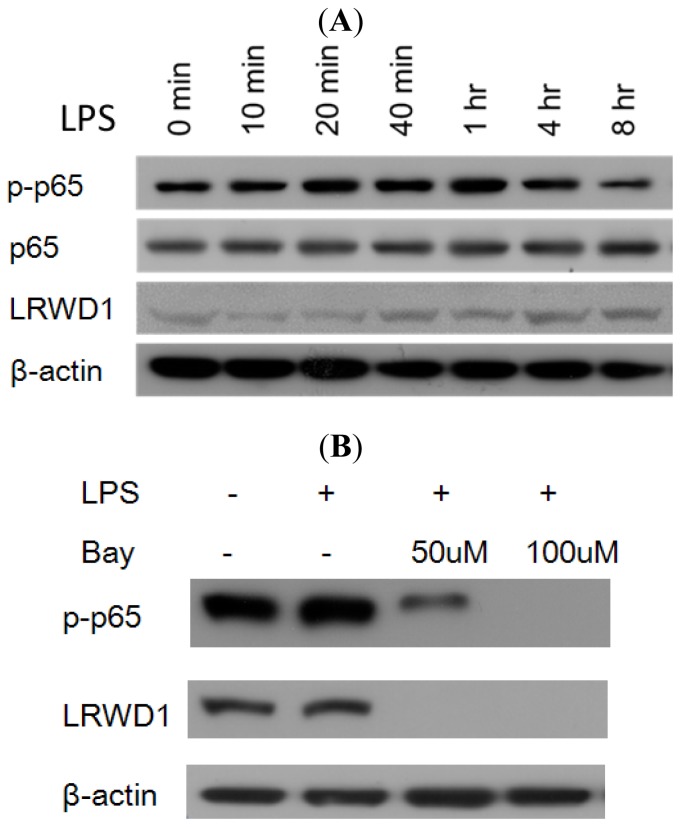

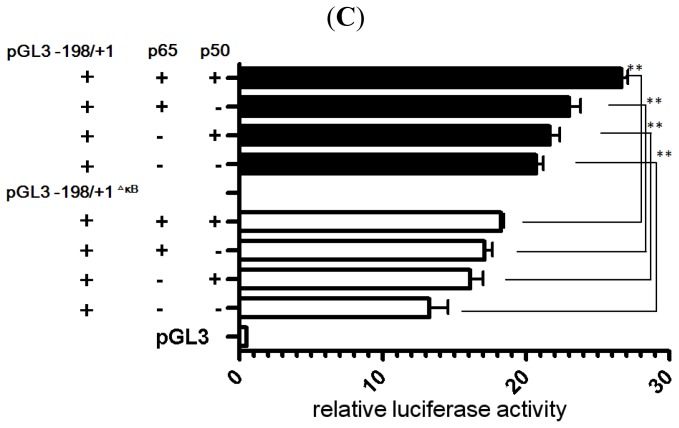
Activation of NF-κB increases *LRWD1* promoter activity. (**A**) lipopolysaccharide (LPS) activates NF-κB and enhances LRWD1 expression. NT2/D1 cells were incubated with LPS (1 μg/mL) for up to 8h as indicated. Cells were lysed and analysed by immunoblotting with anti-p-p65 (s536), anti-p65, or anti-LRWD1 antibody. For normalization, the same blot was probed with the anti-β-actin antibody; (**B**) Inhibition of NF-κB decreases LRWD1 expression. NT2/D1 cells were pretreated with Bay 11–7082 for 1 h, and then stimulated with LPS for 2 h. Whole cell lysates were immunoblotted with an antibody against phospho-p65, LRWD1, or β-actin; (**C**) Overexpression of NF-κB subunits enhances *LRWD1* promoter activity. The KB site (NF-κB binding site) is important for *LRWD1* promoter activity. NT2/D1 cells were transiently transfected with luciferase reporter plasmids driven by either no promoter (pGL3 Basic), or a 198 bp segment of the human *LRWD1* promoter including (pGL3 −198/+1) or lacking (pGL3 −198/+1^ΔκB^) the κB site. The cells were also cotransfected with NF-κB expression vectors or empty expression vectors as indicated. Luciferase activities were normalized to pRL-TK reporter activities and shown as -fold induction compared with the empty vector control. Means ± SD from three independent experiments were shown. * *p* < 0.05; ** *p* < 0.01.

We hypothesized that reducing NF-κB activity might decrease *LRWD1* transcription whereas enhancing NF-κB activity should increase *LRWD1* expression. To test this, we treated NT2/D1 cells with lipopolysaccharide (LPS), a well-characterized activator of NF-κB [10], and monitored its effects on the expression of LRWD1. The level of activated NF-κB (p-p65) in the cells increased significantly 20 min after the addition of LPS and there were higher levels of LRWD1 four to eight hours after the incubation with LPS (Figure 3A). These western blotting results provide evidence that LPS stimulates endogenous *LRWD1* gene expression in NT2/D1 cells. Furthermore, preincubation of the cells with 50 μM or 100 μM BAY 11-7082 which selectively and irreversibly inhibits NF-κB activation for 1 h treatment, then incubates with LPS for 2 h completely attenuated the stimulating effect of LPS on LRWD1 expression, and no increase in the levels of p-p65 and LRWD1 were observed (Figure 3B). Taken together, these data show that LRWD1 expression in NT2/D1 cells increases in response to LPS stimulation through NF-κB activation.

### 2.6. NF-κB Stimulates LRWD1 Promoter Activity

The ability of p50 and p65 (RelA) proteins in the nuclei of NT2/D1 cells to interact with the *LRWD1* promoter suggested that *LRWD1* transcription might be regulated in these cells by NF-κB proteins. To test this hypothesis, pGL3 Basic, pGL3 −198/+1, or pGL3 −198/+1^ΔkB^ was transiently transfected into NT2/D1 cells together with the empty expression vector, a p65 expression vector, or expression vectors for both p65 and p50 (Figure 3C). pCMV4 p65 vector alone or co-expression of pCMV4 p65 vector and pCMV4 p50 vector stimulated LRWD1 activity greater than 2-fold. Removal of the κB site prevented either p65 alone or p65/p50 from significantly stimulating the *LRWD1* promoter. Taken together, these data indicate that full basal *LRWD1* promoter activity requires the conserved NF-κB binding site, and that the NF-κB subunit p65 regulates *LRWD1* transcription in NT2/D1 cells.

## 3. Experimental Section

### 3.1. LRWD1 Expression Assay by RT-PCR

The Human Total RNA Panel was purchased from Clontech (Palo Alto, CA, USA). For the synthesis of cDNA, 12-microliter (μL) aliquots of a master mixture containing 100 nanograms (ng) of RNA, 1 μL of 500 ng/μL oligo(dT)12–18 primer (Invitrogen, Grand Island, NY, USA), and 9 μL of diethylpyrocarbonate-treated water were heated to 70 °C for 10 min and then put on ice. Reverse transcription (RT) reactions were performed in 20-μL volumes containing the master mixture, 4 μL of 5× first strand synthesis buffer, 0.1 M dithiothreitol, 10 mM of each deoxyribonucleotide triphosphate (dNTP), and 200 units of Superscript II RNase H- reverse transcriptase (Gibco/BRL, a division of Invitrogen). The RT temperature profile used was 42 °C for 1 h, 75 °C for 15 min, and final cooling to 4 °C. Aliquots of the complementary DNA (cDNA) were stored at −20 °C until use. The specific primer sequences for human LRWD1 and glyceraldehyde 3-phosphate dehydrogenase (GAPDH) were as follows: LRWD1 forward 5′-TGTGCGTAATTGATTGCC-3′ and reverse 5′-ATCCGCTTGTCATAGG-3′; GAPDH forward 5′-TGAAGGTCGGAGTCAACGGATT-3′ and reverse 5′-CCTGGAAGATGGTGATGG GATT-3′. The polymerase chain reaction (PCR) reaction consisted of each primer set, 1× PCR buffer, 50 mM MgCl_2_, 1.25 mM dNTP, 1 μL of cDNA, and 1 unit of Taq DNA polymerase in a total volume of 20 μL. The reactions were perform in the OmniGene Thermal Cycler™, (Hybaid Ltd., Ashford, UK) for 10 min at 95 °C for initial denaturation, followed by 36 cycles of denaturation at 94 °C for 1 min annealing at 62 °C for 1 min extension at 72 °C for 1 min and a final extension at 72 °C for 10 min. The reaction products were fractionated on 2.5% agarose gels and visualized after staining with ethidium bromide.

### 3.2. Cell Culture and Isolation of Genomic DNA

The NT2/D1 cell line (ATCC number CRL-1973, a pluripotent human testicular embryonal carcinoma cell line derived from a 22 year-old Caucasian man) was obtained from the American Type Culture Collection (ATCC). The cells were grown in 90% Dulbecco’s modified Eagle’s medium supplemented with 4 mM l-glutamine, 1.5 g/L sodium bicarbonate, 4.5 g/L glucose, 10% fetal bovine serum, 100 U/mL penicillin, and 100 μg/mL streptomycin in a humidified atmosphere at 37 °C and 5% CO_2_. Genomic DNA was extracted from NT2/D1 cells using the Puregene DNA isolation kit (Gentra Systems, Minneapolis, MN, USA).

### 3.3. Sources of Plasmids and Plasmid Construction

pCMV4 p65 vector (Addgene plasmid 21966) and pCMV4 p50 vector (Addgene plasmid 21965) were purchased from Addgene, Cambridge, MA, USA. Reporter plasmids were constructed by inserting various lengths of the 5′-upstream region of the human *LRWD1* gene between the *Nhe*I and *Hin*dIII sites of the firefly luciferase reporter pGL3-Basic vector lacking the promoter (Promega Corp., Madison, WI, USA). An approximately 1.3-kb fragment of the *LRWD1* promoter spanning nucleotides −1270 to +1 (−1270/+1) was subcloned into the pGL3-Basic vector. Deletion constructs (−1270/+1, −827/+1, −515/+1, −198/+1, and +1/−1270) were generated by PCR. The pRL-TK vector (Promega Corp., Madison, WI, USA) containing the Renilla luciferase gene under the control of the thymidine kinase promoter was used as a control. The fidelity of all constructs created was confirmed by nucleotide sequence analysis.

### 3.4. Transient Transfection and Luciferase Reporter Gene Assay

For the reporter gene assay, 1 × 10^5^ cells were seeded into 2 mL medium containing 7% (*v*/*v*) FBS in each well of a 6-well culture plate, and cultured for 24 h. The cells were transfected with 0.5 μg each of the reporter plasmid and pRL-TK vector (Promega Corp., Madison, WI, USA) per well using Lipofectamine (Invitrogen, Eugene, OR, USA) according to the manufacturer’s manual, except that the volume was quadrupled. About 48 h later, the cells were washed with phosphate-buffered saline [10 mM sodium phosphate buffer (pH 7.2), 137 mM NaCl, 3 mM KCl] (PBS) and lysed with Lysis buffer (Promega Corp., Madison, WI, USA). After centrifugation at 12,000 × *g* for 10 min at 4 °C, aliquots (20 μL) of the supernatant were used for the measurement of firefly and Renilla luciferase activities according to the manufacturer’s instruction (Promega Corp., Madison, WI, USA). Reporter activity was normalized by calculating the ratio of firefly/Renilla values. Data were analysed using the GraphPad Prism 5.0 statistical software (GraphPad Software, San Diego, CA, USA).

### 3.5. Western Blot Analysis

The cell lysates were collected with RIPA lysis buffer (50 mM Tris-HCl pH 7.4. 150 mM NaCl. 1% NP40. 0.25% Na-deoxycholate. 1 mM PMSF, 1 mM EDTA; 5 mg/mL Aprotinin) containing protease inhibitors (1 mM PMSF, 1 mM orthovanadate, 1 mM EDTA, and 10 mg/mL: leupeptin). Protein concentrations of cell lysates were measured using a Micro BCA protein assay reagent kit (Pierce, Rockford, IL, USA). To the cell lysate, the same volume of SDS-PAGE sample loading buffer [100 mM Tris-HCl, 4% SDS, 5% β-mercaptoethanol, 20% glycerol, and 0.1% bromphenol blue (pH 6.8)] was added, and the cell lysates were boiled for 10 min. Equal amounts of proteins were resolved in 10% SDS-polyacrylamide gels and transferred to PVDF membranes (Millipore, Bedford, MA, USA) using a BioRad transfer system at 110 V for 1 h. The blotted membrane was washed twice with TBS containing 0.1% Tween 20 (TBST; 10 mM Tris-HCl, pH 7.5, 150 mM NaCl, 0.05% Tween-20) and probed with polyclonal anti-LRWD1, p65 (Santa Cruz Biotechnology, Santa Cruz, CA, USA), or Monoclonal p-Ser536-p65 (Cell Signaling, Beverly, MA, USA) sera followed by incubation with a 1:5000 dilution of the horse anti-rabbit IgG, conjugated with peroxidase secondary antibody (Invitrogen, Eugene, OR, USA) in the wash buffer. The filters were then washed several times, and chemiluminescent method was used for detection (SuperSignal West Pico Substrate; Pierce). A monoclonal antibody to β-actin (Sigma, St Louis, MO, USA) was used as controls in Western blot analysis.

### 3.6. Chromatin Immunoprecipitation (ChIP)

NF-κB binding to the *LRWD1* promoter was assessed using the ChIP-IT™ Express Magnetic Chromatin Immunoprecipitation Kits (Active Motif, California, USA) according to the manufacturer’s instruction. Chromatin prepared from NT2/D1 cells was sheared to fragments of 200~500 bp long and immunoprecipitated with an anti-NF-κB polyclonal antibody (Cell Signaling, Beverly, MA, USA) or an IgG control antibody (Sigma, St. Louis, MO, USA). The immunoprecipitated DNA was purified and subjected to PCR amplification of 268 bp fragments ((−275)~(−8)) of the *LRWD1* promoter and a 250 bp fragment ((−5895)~(−5646)) about 6 kb upstream of the *LRWD1* promoter as negative control. The sequences of PCR primers were: CHIP-hLRWD1-F: 5′ GATGCAGAGCGACGTTTGT3′ ((−275)~(−257)); CHIP-hLRWD1-R: 5′ AGGAAGCGGAACCCAGGC3′ ((−25)~(−8)); up6000-hLRWD1-F: 5′AGCCAGAAGAATGGC TTGAA3′ ((−5895)~(−5876)); and up6000-hLRWD1-R: 5′ ATTGTCACCATCCGTGTCCT3′ ((−5665)~(−5646)). The PCR amplification cycle consisted of an initial denaturation step at 95 °C for 45 s, followed by 30 cycles of denaturation at 95 °C for 30 s, annealing at 60 °C for 30 s, extension at 72 °C for 60 s, and a final extension step at 72 °C for 10 min. The PCR products were analyzed by electrophoresis on 2% agarose gels and their sequences were confirmed using an automatic sequencer (ABI 377, Applied Biosystems/PE).

### 3.7. Gel Electrophoretic Mobility Shift Assay (EMSA)

Preparation of nuclear extracts and EMSA were performed using the LightShift^®^ Chemiluminescent EMSA Kit (Pierce, Rockford, IL, USA) according to the manufacturer’s instruction. The double-stranded DNA probes contained sequences corresponding to −143 to −115 of the *LRWD1* promoter. The sequences for the wild-type and mutant probes were 5′GAAGGACTTCGGGGGTCTCGGCGGCAGCC3′ and GAAGGACTTCGAAAAGAGCGGCGGCAGCC-3′, respectively, with the wild-type and mutated NF-κB binding sites underlined. The probes were end-labeled with biotin (Mission Biotech Co., Ltd., Taipei, Taiwan). The DNA binding reaction was performed according to the manufacturer’s instruction. For cold competition, nuclear extracts were incubated with unlabelled wild-type or mutant duplex oligonucleotides for 15 min prior to the addition of labeled probes. DNA/protein complexes were resolved on a 5% polyacrylamide gel and analyzed according to the manufacturer’s instruction.

### 3.8. Statistical Analysis and Bioinformatics Software

The significance levels for all statistical tests were 0.05 by the GraphPad Prism software (http://www.graphpad.com/prism/prism.htm) [25]. Putative transcription factor binding sites in the promoter region of *LRWD1* were identified using the Promoter 2.0 Prediction Server (http://www.cbs.dtu.dk/services/Promoter/) [24] and Searching Transcription Factor Binding Sites (TFSEARCH, http://www.cbrc.jp/research/db/TFSEARCH.html) [26].

## 4. Discussion and Conclusions

The LRWD1 5′-flanking sequence has the features of a TATA-less and GC1 boxes containing promoter, and contains consensus binding sites for several transcription factors, including NF-KB [6], Sp1 [8,27], and AP-2 [28], that may be functionally important. TATA-less promoters have been found in a variety of genes, including housekeeping [29] and TNF-receptor family genes [30], that usually have variable transcription start sites and are ubiquitously expressed in multiple tissues. The promoter of testis H1-like histone H1LS1 gene is TATA-less and testis-specific [31]. The transcription level of the mPAB II gene in testis increases during spermatogenesis, and the mPAB II protein accumulates densely in the nuclei of spermatocytes and round spermatids. The promoter region of mPAB II contains a CpG island but lacks a TATA box, suggesting that the mPAB II mRNA is transcribed by a TATA-less promoter [32]. CCAAT boxes have been found in the promoters of other TATA-less, testis-specific genes, including rat and rabbit histone H2B genes [33], human and mouse PGK2 genes [34,35], and mouse Ldh-c [36]. Sp1 sites (GC1 boxes) have also been observed in the core promoters of other TATA-less testis-specific genes such as Pdha-2 [37], human LDH-c [38], human and murine Pgk-2 [39], and Prm-2 (protamine-2) [40]. The isolation of two testis-specific isoforms of SP1 [41], and the higher level of Sp1 expression found in spermatids [42] may reflect a particular role for this transcription factor in relation to the expression of genes possessing TATA-less promoters during spermatogenesis. Meanwhile, we have shown that a 198 bp segment of the TATA-less core promoter region of the LRWD1 gene has specific promoter activity. Our transient transfection assay using various deletion mutants identified a segment from −198 to +1 that contains the basal promoter activity of the LRWD1 gene. In addition, the sequence of the human LRWD1 promoter region is conserved in great apes (Figure 1D), but not in other mammals or vertebrates such as mouse, rat, or zebrafish. We suggest that NF-κB is an important regulator of LRWD1 expression.

NF-κB is a well-known redox regulated transcription factor that has been suggested to play a crucial role in spermatogenesis. In human seminiferous tubules, RelA is present mainly in the cytoplasm of spermatogonia and early meiotic germ cells [15]. In our previous study, LRWD1 was detected most abundantly in the cytoplasm of spermatocytes, spermatids, and the centrosome of spermatozoa (manuscript submitted). We suggest that a small fraction of NF-κB is present as the phosphorylated active form in the nuclei of spermatocytes and spermatids and binds to the LRWD1 promoter to regulate LRWD1 expression during spermatogenesis. So far few studies focused on specific target genes regulated by NF-κB in human testes. In this study, we have identified LRWD1 as a transcriptional target of NF-κB in human testis cells (Figure 1B). Inhibition or activation of NF-κB resulted in decreased or increased LRWD1 expression (Figure 3). The LRWD1 promoter activity was positively regulated by NF-κB, and this regulation was dependent on the presence of a conserved κB site in the LRWD1 promoter region (Figure 3C). Chromatin immunoprecipitation analysis and EMSA showed basal binding of the p65 subunit of NF-κB to the LRWD1 promoter region encompassing the κB site, which is enhanced after the activation of NF-κB by lipopolysaccharide. Over-expression of NF-κB subunits in cells enhanced LRWD1 promoter activity (Figure 3). Our results suggest that the core promoter of LRWD1 is an important factor for its testis-specific expression. Based on these data, we hypothesize that NF-κB is an important regulator for the expression of the testis-enriched LRWD1 gene.
